# Supplementary material for: Comparing BeadChip and WGS Genotyping: Non-Technical Failed Calling Is Attributable to Additional Variation within the Probe Target Sequence
Source: Genes (Basel). 2022 Mar 9;13(3):485. doi: 10.3390/genes13030485 (PMC8948885; doi:10.3390/genes13030485)
Supplement: Supplementary file 1 [file genes-13-00485-s001.zip › FigureS1.pdf]

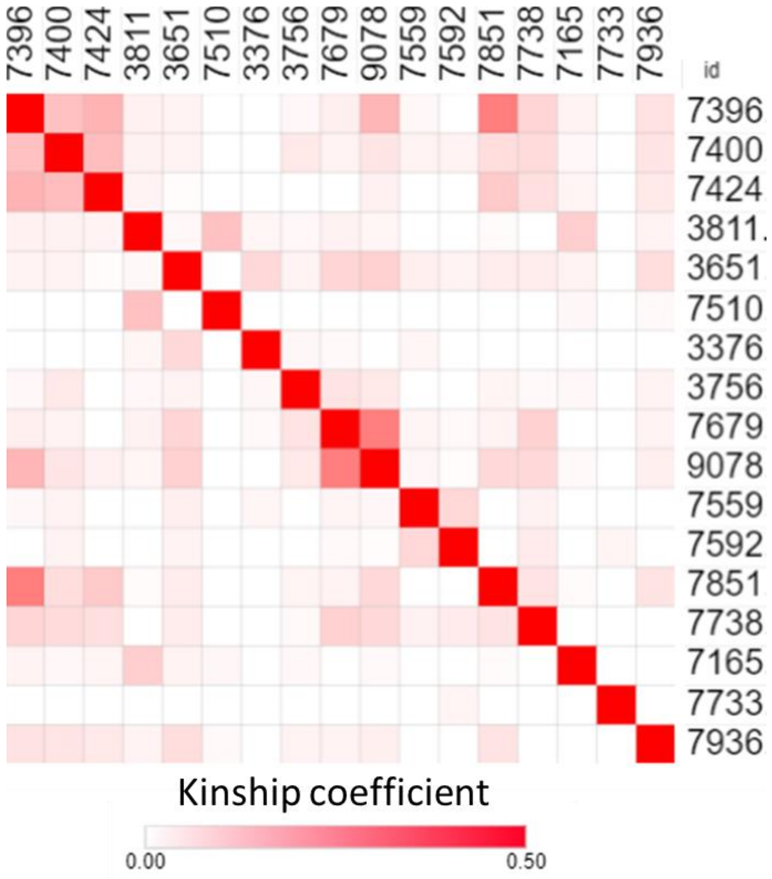

Figure S1. Heatmap of the clustered kinship matrix of 17 Israeli Holstein bulls that underwent whole-genome sequencing. A total of 42,848 informative SNPs were used to calculate the kinship coefficient for all possible pairs of bulls using the KING software [https://doi.org/10.1093/bioinformatics/btq559]. A custom-designed Perl script was further used to create a symmetric matrix of the bulls' kinship scores. X- and Y- axes denote the bulls' identifiers. Red color intensities mark the kinship coefficient of each pair, indicating the kinship level, where 0 indicates remoted or no kinship, 0.25 indicates kinship from the 1st degree, whereas, the maximum of 0.5 denotes total identity on the diagonal.
